# Moderate Fluid Shear Stress Regulates Heme Oxygenase-1 Expression to Promote Autophagy and ECM Homeostasis in the Nucleus Pulposus Cells

**DOI:** 10.3389/fcell.2020.00127

**Published:** 2020-03-03

**Authors:** Sheng Chen, Lei Qin, Xiaohao Wu, Xuekun Fu, Sixiong Lin, Di Chen, Guozhi Xiao, Zengwu Shao, Huiling Cao

**Affiliations:** ^1^Department of Orthopaedics, Union Hospital, Tongji Medical College, Huazhong University of Science and Technology, Wuhan, China; ^2^Guangdong Provincial Key Laboratory of Cell Microenvironment and Disease Research, Shenzhen Key Laboratory of Cell Microenvironment, and Department of Biology, Southern University of Science and Technology, Shenzhen, China; ^3^Guangdong Provincial Key Laboratory of Orthopedics and Traumatology, Orthopedic Research Institute and Department of Spinal Surgery, The First Affiliated Hospital of Sun Yat-sen University, Guangzhou, China; ^4^Department of Orthopedic Surgery, Rush University Medical Center, Chicago, IL, United States

**Keywords:** nucleus pulposus cell, fluid shear stress, autophagy, heme oxygenase-1, intraflagellar transport (IFT) 88

## Abstract

In vertebrate, the nucleus pulposus (NP), which is an essential component of the intervertebral disk, is constantly impacted by fluid shear stress (FSS); however, molecular mechanism(s) through which FSS modulates the NP homeostasis is poorly understood. Here we show that FSS regulates the extracellular matrix (ECM) homeostasis in NP cells. A moderate dose of FSS (i.e., 12 dyne/cm^2^) increases the sulfated glycosaminoglycan (sGAG) content and protein levels of Col2a1 and Aggrecan and decreases those of matrix metalloproteinase 13 (MMP13) and a disintegrin and metalloproteinase with thrombospondin motif 5 (ADMATS5) in rat NP cells, while a higher dose of FSS (i.e., 24 dyne/cm^2^) displays opposite effects. Results from RNA sequencing analysis, quantitative real-time RT-PCR analysis and western blotting establish that the heme oxygenase-1 (HO-1) is a key downstream mediator of the FSS actions in NP cells. HO-1 knockdown abolishes FSS-induced alterations in ECM protein production and sGAG content in NP cells, which is reversed by HO-1 induction. Furthermore, FSS activates the autophagic pathway by increasing the LC3-II/LC3-I ratio, Beclin-1 protein level, and formation of autophagosome and autolysosome and thereby regulates ECM protein and sGAG production in a HO-1 dependent manner. Finally, we demonstrate that the intraflagellar transport (IFT) 88, a core trafficking protein of primary cilia, is critically involved in the HO-1-mediated autophagy activation and ECM protein and sGAG production in FSS-treated NP cells. Thus, we for the first time demonstrate that FSS plays an important role in maintaining ECM homeostasis through HO-1-dependent activation of autophagy in NP cells.

## Introduction

Low back pain is a common and frequently recurring musculoskeletal disorder, which causes heavy financial burden worldwide (Chen et al., 2017; Clouet et al., 2018; Ma et al., 2019). Epidemiological investigations show that over 80% of the world population will experience LBP at some point in their life, and LBP has become the leading cause of years lived with disability and the second most common reason for hospital visits (Maher et al., 2017; Henry et al., 2018; Chen et al., 2019). In the United States, the annual cost of LBP is over $100 billion, which is even more than the total cost of stroke, coronary artery disease, diabetes, rheumatoid disease and respiratory infection (Katz, 2006). Intervertebral disk degeneration is considered to be the main cause of LBP (Frapin et al., 2019), thus, it is of great importance to understand the pathophysiology of IVD.

The IVD is an avascular and fibrocartilaginous tissue, which lies between the vertebral bodies and functions as a shock absorber by distributing biomechanical loads along the spinal column (Priyadarshani et al., 2016). Each IVD can be roughly divided into three distinct regions: in the center was the gelatinous NP, encompassed by outer fibrocartilaginous AF, bordered superiorly and inferiorly by CEP (Sakai and Grad, 2015). Under normal physiological condition, although there is low cellularity in both NP and AF tissues, the resident cells, especially NP cells, synthesize and secrete the complex ECM molecules to maintain ECM homeostasis and resist the mechanical stresses, which plays a key role in the protection against IVD degeneration (Hu et al., 2016; Silagi et al., 2018; Bonnevie et al., 2019). In healthy NP tissues, ECM contains three major components: collagen type II (Col2), glycosaminoglycan (GAG) and PGs. Aggrecan, bound by massive sGAG side chains, is the most abundant PG in NP. During the process of IVD degeneration, the degradation of these ECM proteins by catabolic proteinases, such as a disintegrin and metalloproteinase with thrombospondin motif 5 (ADMATS5) and matrix metalloproteinase 13 (MMP13), is accelerated, while their biosynthesis is decreased, leading to an imbalance between the anabolic and catabolic metabolism of ECM.

**FIGURE 1:**
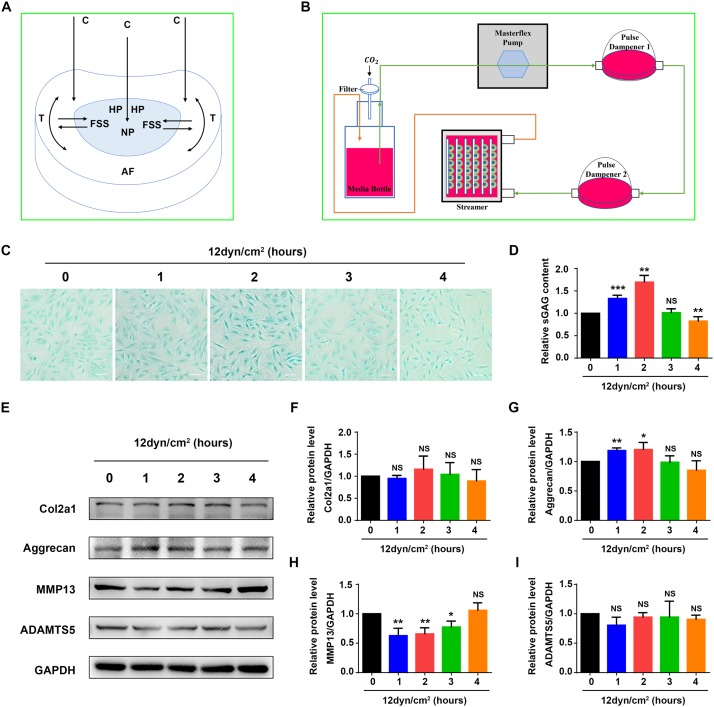
Fluid shear stress regulates expression of ECM proteins and sGAG content in nucleus pulposus cells. **(A)** The formation of fluid shear stress (FSS) in nucleus pulposus cells (NP). Compressive stress (C), hydrostatic pressure (HP), and tensile stress (T) are indicated. **(B)** Schematic diagram of the Flexcell Streamer System. **(C)** Alcian blue staining. NP cells were treated with 12 dyne/cm^2^ FSS for the indicated times. Bar, 100 μm. **(D)** sGAG content. NP cells were treated as in panel **(B)**, followed by the Blyscan Sulfated Glycosaminoglycan Assay. **(E–I)** Western blotting. NP cells were treated as in panel **(C)**, followed by Western blotting analyses for expression of Col2a1, aggrecan, MMP13, and ADAMTS5. Quantitative data from three independent experiments **(D,F–I)**. Results are expressed as mean ± standard deviation (s.d.). NS, no statistical significance, *P < 0.05, **P < 0.01, and ***P < 0.001 vs. un-treated group (0 h).

Increasing evidence suggests that mechanical stresses, including FSS, hydrostatic pressure, compressive stress, tensile stress and other mechanical stresses, play a pivotal role in the regulation of ECM homeostasis in IVD (Neidlinger-Wilke et al., 2014; Chou et al., 2016). To maintain erect posture, the IVD is under compressive stress, which can induce deformation and produce hydrostatic pressure in NP. The hydrostatic pressure is then transferred to AF and induces the generation of tensile stress. During body motion and spine movement, tissue fluid within the NP flows in and out to accommodate the change of these stresses. Consequently, NP cells are constantly exposed to FSS (Figure 1A; Ye et al., 2018). However, whether and how FSS regulates ECM homeostasis in NP cells is poorly understood.

Autophagy is a highly conserved and adaptive process involving selectively eliminating and recycling bulk harmful cytoplasmic materials, such as misfolded proteins and damaged intracellular organelles, thereby acting as a main cytoprotective system to maintain cellular homeostasis (Towers and Thorburn, 2016; Dikic and Elazar, 2018). Generally, cells exhibit a low and basal level of autophagy. But the level of autophagy can significantly increase in response to external environment stress, including mechanical stress and nutrient deprivation, in order to provide nutrients for essential cellular functions (Gross and Graef, 2019). The HO-1, which has been identified in many tissues and organs as well as different pathophysiological scenarios, is the rate-limiting enzyme in the metabolism of heme into biliverdin, carbon monoxide and iron, and can exert cytoprotective effects against various external environment stress-induced oxidative stress, inflammation and cell death (Otterbein et al., 2016; Chiang et al., 2018). Using our recently established rat NP cell line and a Flexcell Streamer System, in the present study we demonstrate that moderate FSS maintains ECM homeostasis by promoting cell autophagy through modulation of HO-1.

## Materials and Methods

### NP Cell Line Culture and FSS Experiments

An immortalized rat NP cell line used in this study was described in Oh et al. (2016). The cells were cultured in Dulbecco’s Modification of Eagle’s Medium (10-013-CVR; Corning, United States) containing 10% FBS (10099-141; Gibco, Australia) supplemented with 1% penicillin-streptomycin (SV30010; Hyclone, United States) at 37°C with 5% CO_2_. FSS experiments were conducted as previously described (Yang et al., 2019). Cells were seeded onto collagen I-coated culture slips (75 mm × 25 mm × 1 mm; FFCS-C; Flexcell, United States) at a density of 3.0 × 10^4^/cm^2^ and incubated in a 5% CO_2_ incubator at 37°C. When cells reached up to 85% confluence, the slips were then placed in a parallel plate flow chamber of Streamer^®^ System (STR-4000; Flexcell, United States) (Figure 1B) and cells are exposed to 12 or 24 dyne/cm^2^ FSS for 0, 1, 2, 3, and 4 h. For certain experiments, NP cells were pre-treated with 10 μM CoPP (Sigma, United States, C1900) for 1 h or 500 nM rapamycin (Selleck, United States, S1039) for 12 h before exposure to FSS.

### RNA Sequencing Analysis

Total RNA was isolated from NP cells with or without FSS stimulation using a TransZol Up Plus RNA Kit (ER501-01; Transgen, China) and 3 μg RNA per sample was used as input material for the RNA sample preparations. Sequencing libraries were generated using NEBNext^®^ UltraTM RNA Library Prep Kit (Illunina, NEB, United States) and the library quality was assessed on the Agilent Bioanalyzer 2100 system. After cluster generation, the library preparations were sequenced on an Illumina Hiseq platform and 150 bp paired-end reads were generated. After quality control, reads mapping to the reference genome and quantification of gene expression level, differential expression analysis was performed by using the DESeq2 R package (1.16.1), and GO and KEGG enrichment analyses were performed by using the cluster Profiler R package. The RNASeq data have been deposited in Sequence Read Archive (SRA, PRJNA587407).

### Analysis of sGAG Content

After fixed with 4% paraformaldehyde, NP cells were dehydrated by different concentrations of ethanol and xylol. The cells were then stained with alcian blue. To measure the sGAG content, cells were digested with papain extraction reagent and then the sGAG content was quantified by Blyscan Sulfated Glycosaminoglycan Assay (B1000; Biocolor, United Kingdom) according to the manufacturer’s instructions.

### Quantitative Real-Time Polymerase Chain Reaction (qRT-PCR) Analysis

Total RNA was extracted from NP cells using Tripure Isolation Reagent (11667165001; Roche, Germany). Reverse transcription (RT) and qRT-PCR analyses were performed as we described previously (Cao et al., 2010). Briefly, RNA was reverse transcribed into cDNA using PrimeScript^TM^ RT Master Mix (RR036A; Takara, Japan) according to the manufacturer’s protocol. After reverse transcription, qRT-PCR was performed using iTaq^TM^ Universal SYBR^®^ Green Supermix (172-5121; Bio-Rad). The 2^–ΔΔ*CT*^ method was used to analyze the data, and GAPDH was used as an internal control. Primer sequences are shown in Supplementary Table S1.

### Western Blotting Analyses

Nucleus pulposus cells were lysed with Tripure Isolation Reagent (11667165001; Roche, Germany). After centrifugation at 12,000 × *g* for 10 min at 4°C, protein pellet was isolated from the lower red organic phase by alcohol precipitation steps. Wash the protein pellet with 0.3 M guanidine hydrochloride/95% ethanol for three times and dissolve the protein pellet by adding 1% sodium dodecyl sulfate. The protein content was detected by Pierce^TM^ BCA Protein Assay Kit (23225; ThermoFisher Scientifc, United States). Western blot analyses were performed as previously described (Cao et al., 2020). Proteins were loaded on sodium dodecyl sulfate-polyacrylamide gel for electrophoresis. After protein transfer, the membranes were blocked by 5% skimmed milk and then incubated overnight at 4°C with antibody against Col2a1 (1:1000, A1560; Abclonal Technology, China), Aggrecan (1:1000, ab36861; Abcam, United Kingdom), MMP13 (1:3000, ab39012; Abcam, United Kingdom), ADAMTS5 (1:500, ab41037; Abcam, United Kingdom), HO-1 (1:1000, 10701-1-AP; Proteintech, China), LC3-I/II (1:1000, ab62721; Abcam, United Kingdom), Beclin-1 (1:1000, 3738s; Cell Signaling Technology, United States), IFT 88 (1:500, 13967-1-AP; Proteintech, China) and GAPDH (1:2000, TA-08; ZSGB-Bio, China). After washing with TBST for three times, the membranes were incubated with secondary antibodies for 1 h at room temperature. Finally, the proteins were detected using the enhanced chemiluminescence method.

### Immunofluorescence

Immunofluorescence staining was performed according to the method we previously used (Cao et al., 2020). After fixation in 4% paraformaldehyde at room temperature for 10 min, cells were permeabilized in 0.1% triton-100 for 15 min and blocked with 1% bovine serum albumin for 1 h at room temperature. Subsequently, cells were incubated overnight at 4°C with antibody against HO-1 (1:200, 10701-1-AP; Proteintech, China) and acetylated-tubulin (1:400, T6793; Sigma, United States), and then incubated for 1 h at room temperature with AlexaFluor 488-conjugated rabbit and mouse secondary antibodies (1:500, A11008 and A11001; ThermoFisher Scientifc, United States). After stained with DAPI, the cells were examined with a confocal microscope (A1R; Nikon, Japan) and analyzed with ImageJ software (Version 1.50; National Institutes of Health, United States).

### Cilium Length and Prevalence Measurements

A Nikon A1R confocal microscope with an oil immersion × 100 objective was used to create maximum projection of confocal z-stacks from which cilia length was measured by ImageJ software (Version 1.50; National Institutes of Health, United States). Confocal z maximum projection was also used to determine the cilium prevalence and DAPI nuclear staining.

### Transfection of Small Interfering RNA (siRNA)

The rat HO-1-siRNAs and IFT88-siRNAs were designed and manufactured by GenePharma (GenePharma Co.,Ltd., China). Transfection was performed as previously described (Cao et al., 2013). Briefly, NP cells were transfected with negative control (NC) siRNA and three independent HO-1-siRNAs and IFT88-siRNAs, respectively, the sequences are shown in Supplementary Table S1. The most effective target sequences for HO-1-siRNA (#2) and IFT88-siRNA (#1) were used. Cells were transfected with siRNAs at a concentration of 50 pmol/10^5^ cell using Lipofectamine^TM^ RNAiMAX Transfection Reagent (13778150; ThermoFisher Scientific, United States). 24 h later, the transfected cells were digested and seeded onto collagen I-coated culture slips for further study.

### Transmission Electron Microscopy (TEM)

Harvested NP cells were washed in PBS and deionized water, and then pelleted by centrifugation. Cells were fixed with 2.5% glutaraldehyde for 2 h and 1% osmium tetroxide for 2 h, respectively. The cells were then dehydrated in ethanol and infiltrated and embedded in Embed 812. Ultrathin sections were stained with UranyLess (22409; EMS, United Kingdom) and Lead Citrate (22410; EMS, United Kingdom) and examined with a TEM (HT7700; Hitachi, Japan).

### Autophagy Detection Using mRFP-GFP-LC3 Lentiviral Vector

Nucleus pulposus cells were seeded in 24 well plates (1 × 10^5^/plate). 24 h later, cells were infected with lentiviral vector (HanBio Technology Co.,Ltd., China) according to the manufacturer’s instructions. 48 h after infection, puromycin was used to screen stable NP cell line expressing mRFP-GFP-LC3. The transfected cells were then used for autophagy detection. Autophagy was determined by confocal microscope (A1R; Nikon, Japan). Autophagic flux was detected by evaluating the number of GFP and mRFP puncta. Free red dots indicated autolysosomes and yellow dots indicated autophagosomes.

### Statistical Analysis

Statistical analysis was performed using GraphPad Prism 6 software (GraphPad Software Inc., United States). All experiments were carried out at least three independent experiments and the data were presented as mean ± standard deviation (s.d.). For analysis of two-group parameters, Student’s *t*-tests were used. Statistical significance was set at a level of *P* < 0.05.

## Results

### FSS Regulates ECM Protein Expression and sGAG Content in Rat NP Cells

As an initial step to explore mechanism(s) through which mechanical force regulates the homeostasis of IVD, we performed experiments to determine the effects of FSS on expression of ECM proteins in rat NP cells. The NP cells were subjected to FSS treatment at a dose of 12 dyne/cm^2^ for the indicated times (Figure 1B), followed by assays for sGAG content and expression of several key ECM proteins. The results showed that sGAG content and aggrecan protein level were significantly increased at 1 and 2 h of FSS treatment, and MMP13 protein level was significantly decreased at 1, 2 and 3 h of FSS treatment (Figures 1C–E,G,H). FSS treatment slightly increased Col2a1 expression and decreased ADAMTS5 expression (Figures 1F,I). However, when NP cells were exposed to 24 dyne/cm^2^ FSS treatment, sGAG content and the protein levels of Col2a1 and aggrecan were decreased, while the levels of MMP13 and ADAMTS5 were increased in a time-dependent manner (Supplementary Figure S1). These results suggest that moderate FSS upregulates sGAG content and anabolic ECM proteins (Col2a1 and aggrecan), and downregulates catabolic ECM proteins (MMP13 and ADAMTS5) in NP cells, while high FSS exerts opposite effects. We focused our next studies to determine potential mechanism(s) through which moderate FSS (i.e., 12 dyne/cm^2^ for 2 h) regulates expression of key ECM proteins and sGAG content in NP cells.

### HO-1 Is Largely Upregulated by Moderate FSS in NP Cells

**FIGURE 2:**
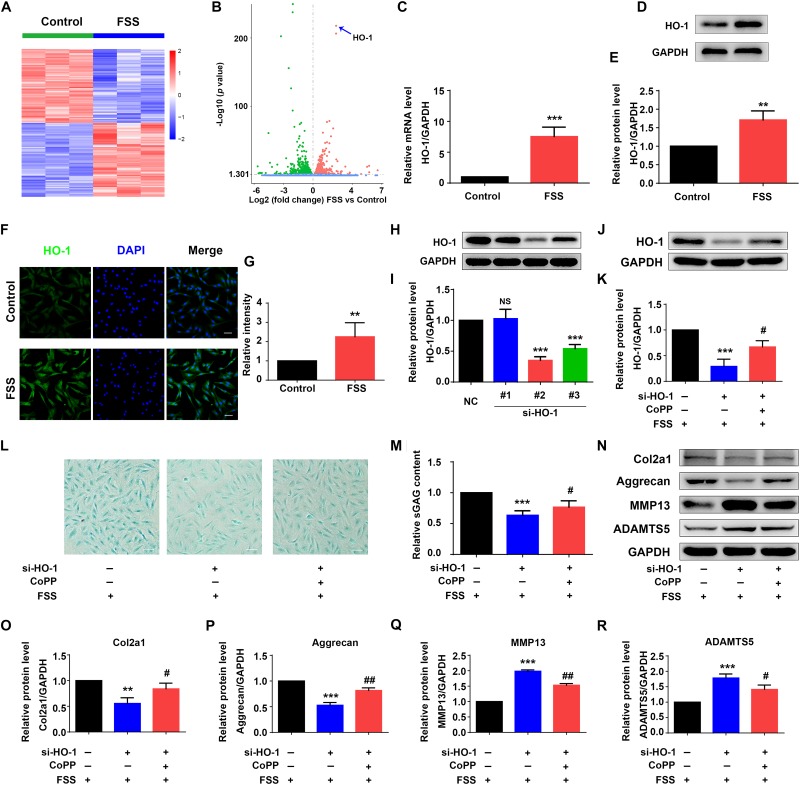
Heme oxygenase-1 regulates FSS-mediated ECM production in NP cells. **(A)** The heat map of RNA-seq data. NP cells were treated with or without 12 dyne/cm^2^ FSS for 2 h, followed by RNA-seq analysis as described in Materials and Methods. **(B)** Volcano plot of RNA-seq data. Blue arrow indicates heme oxygenase-1 (HO-1). **(C)** Quantitative real-time reverse transcriptase-polymerase chain reaction (qRT-PCR) analysis. NP cells were treated with or without 12 dyne/cm^2^ FSS for 2 h, followed by qRT-PCR analysis. **(D,E)** Western blotting. NP cells were treated as in panel **(C)**, followed by western blotting. Quantitative data from three independent experiments **(E)**. **(F,G)** Immunofluorescence (IF) staining. NP cells were treated as in panel **(C)**, followed by IF staining **(E)**. Bar, 50 μm. Quantitative data from three independent experiments **(G)**. **(H,I)** HO-1 siRNA knockdown. NP cells were transfected with negative control siRNA (NC-siRNA) and three HO-1-siRNA (#1, #2, #3). Then, cells were subjected to western blotting for HO-1 **(H)**. Quantitative data from three independent experiments **(I)**. Note: #2 HO-1-siRNA showed the best knocking down effect and was used for all following experiments in this study. **(J,K)** Western blotting. NP cells were transfected with negative control siRNA (NC-siRNA) and HO-1-siRNA (#2). Then, cells were pretreated with or without HO-1 inducer cobalt protoporphyrin IX (CoPP) (10 μM) and subjected to 12 dyne/cm^2^ FSS for 2 h, followed by western blotting for HO-1 **(J)**. Quantitative data from three independent experiments **(K)**. **(L,M)** sGAG content. NP cells were treated as in panel **(J)**, followed by alcian blue staining and Blyscan Sulfated Glycosaminoglycan Assay. Bar,100 μm. Quantitative data of sGAG content from three independent experiments **(M)**. **(N–R)** Western blotting. NP cells were treated as in panel **(J)**, followed by western blotting for expression of the indicated proteins. Quantitative data from three independent experiments **(O–R)**. Results are expressed as mean ± standard deviation (s.d.). NS, no statistical significance, **P < 0.05, ***P < 0.001 vs. control, NC or FSS + NC-siRNA group; ^#^*P* < 0.01 and ^##^*P* < 0.01 vs. FSS + HO-1-siRNA group.

To identify genes that were regulated by moderate FSS treatment, NP cells were treated with or without FSS (12 dyne/cm^2^ for 2 h). Total RNAs from both groups were isolated and subjected to RNA sequencing analysis as described in Materials and Methods. Genes upregulated (1463) or downregulated (1467) were presented in Supplementary Table S2. Among the upregulated genes, we found that HO-1 was significantly upregulated by FSS treatment with the lowest *p* value (Figures 2A,B). Results from qRT-PCR analysis, western blotting and immunofluorescence staining further verified that the levels of HO-1 mRNA and protein were dramatically increased by FSS treatment (Figures 2C–G).

### HO-1 Is Critical for Moderate FSS Regulation of ECM in NP Cells

Since HO-1 was reported to regulate ECM metabolism in NP cells (Hu et al., 2016), we next determined whether HO-1 is involved in FSS regulation of ECM proteins in NP cells. To do this, NP cells were transfected with negative control siRNA (NC-siRNA) and three different HO-1 siRNAs (#1, #2, and #3). We chose the #2 HO-1 siRNA for HO-1 knocking down in this study since the #2 HO-1 siRNA showed the best knocking down efficiency (Figures 2H,I). To determine whether HO-1 is involved in FSS regulation of ECM, NP cells were first transfected with control siRNA or HO-1-siRNA (#2). Then, cells were pretreated with or without HO-1 inducer cobalt protoporphyrin IX (CoPP) (10 μM) for 1 h and subjected to 12 dyne/cm^2^ FSS for 2 h. As expected, HO-1 siRNA dramatically decreased the level of HO-1 protein, which was markedly reversed by treatment of CoPP treatment (Figures 2J,K). Furthermore, HO-1 siRNA significantly reduced the synthesis of sGAG, which was partially reversed by CoPP treatment (Figures 2L,M). Similarly, results from western blot analyses revealed that HO-1 siRNA downregulated the levels of anabolic ECM proteins (Col2a1 and aggrecan) and upregulated the levels of catabolic ECM proteins (MMP13 and ADAMTS5), which could be reversed by CoPP treatment (Figures 2N–R).

### FSS Promotes Autophagy in NP Cells

**FIGURE 3:**
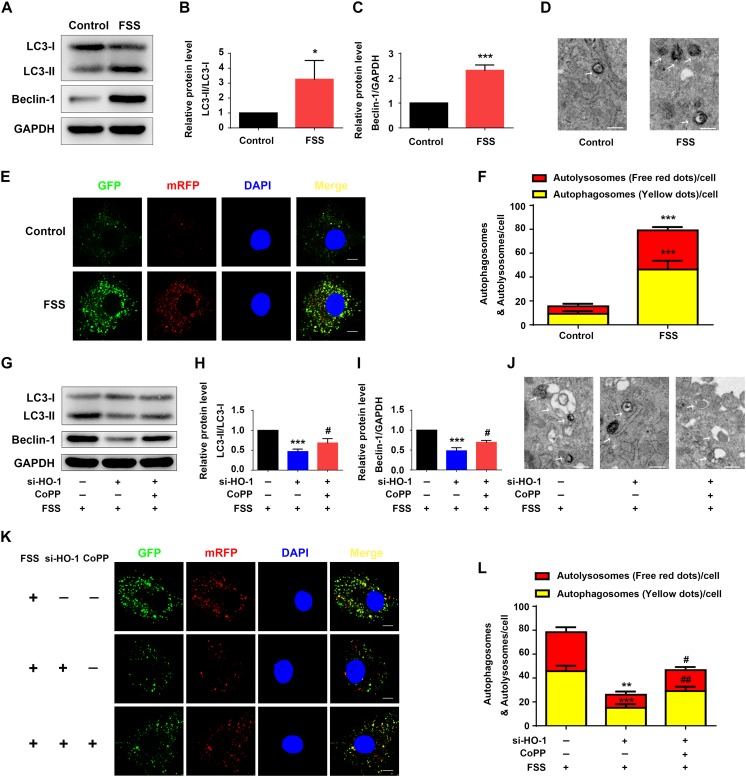
HO-1 mediates FSS-induced autophagy in NP cells. **(A–C)** Western blotting. NP cells were treated with or without 12 dyne/cm^2^ FSS for 2 h, followed by western blotting for expression of the indicated proteins. Quantification of LC3-II/LC3-I ratio **(B)** and Beclin-1 **(C)**. **(D)** Transmission Electron Microscopy (TEM). NP cells were treated as in panel **(A)**. TEM images are shown. Arrows indicate double-membrane autophagosomes and autolysosomes at various stages. Bar, 500 nm. **(E,F)** Measurements of autophagosomes and autolysosomes. NP cells were infected with lentivirus expressing a tandem mRFP-GFP-LC3 construct and treated with or without 12 dyne/cm^2^ FSS for 2 h. Fluorescence images were obtained. Bar, 5 μm. Quantification of autophagosomes (yellow dots) and autolysosomes (free red dots) **(F)**. **(G–I)** Western blotting. NP cells were first treated with and without HO-1 siRNA knockdown and in the presence and absence of CoPP. Then, cells were pretreated with or without HO-1 inducer cobalt protoporphyrin IX (CoPP) (10 μM) and subjected to 12 dyne/cm^2^ FSS for 2 h, followed by western blotting for expression of the indicated proteins. Quantification of LC3-II/LC3-I ratio and Beclin-1 from three independent experiments **(H,I)**. **(J)** TEM. NP cells were treated as in panel **(G)**. TEM images are shown. The arrows indicate double-membrane autophagosomes and autolysosomes. Bar, 500 nm. **(K,L)** Measurements of autophagosomes and autolysosomes. NP cells were transduced with lentivirus expressing a tandem mRFP-GFP-LC3 construct and treated as in panel **(G)**. Fluorescence images were obtained. Bar, 5 μm. Quantification of autophagosomes (yellow dots) and autolysosomes (free red dots) from three independent experiments **(L)**. *P < 0.05, **P < 0.01, and ***P < 0.001 vs. control; ^#^*P* < 0.05 and ^##^*P* < 0.01 vs. FSS + HO-1-siRNA group.

Fluid shear stress activates autophagy in multiple cell types (Zhang et al., 2018; Yang et al., 2019). Thus, we determined whether FSS activates autophagy in NP cells. Results showed that NP cells treated with FSS displayed increased autophagy compared to untreated cells, as demonstrated by dramatic increases in the LC3-II/LC3-I ratio and Beclin-1 protein level (Figures 3A–C). Likewise, TEM analyses showed a marked increase in the numbers of autophagosome and autolysosome in FSS-treated cells relative to untreated cells (Figure 3D). Furthermore, immunofluorescence confocal microscopy analyses revealed that the numbers of both autophagosome (yellow dots) and autolysosome (free red dots) were dramatically increased in FSS-treated NP cells compared to those in untreated cells (Figures 3E,F).

### HO-1 Regulates the FSS-Induced Autophagy in NP Cells

Heme oxygenase-1 was reported to activate autophagy in NP cells (Hu et al., 2016). Thus, we next determined whether HO-1 plays a role in mediation of the FSS-induced autophagy in NP cells. NP cells were treated with FSS, with or without CoPP treatment, in the presence or absence of HO-1 knockdown by siRNA. The results showed that HO-1 knockdown dramatically reduced the increases of LC3-II/LC3-I ratio and Beclin-1 expression in NP cells induced by FSS treatment, which was markedly reversed by CoPP treatment (Figures 3G–I). TEM analyses showed that HO-1 siRNA significantly reduced the numbers of autophagosome and autolysosome in FSS-treated NP cells compared to control siRNA/FSS-treated cells, which was largely reversed by CoPP treatment (Figure 3J). Similarly, immunofluorescence confocal microscopy analyses showed that the numbers of both autophagosome (yellow dots) and autolysosome (free red dots) were dramatically decreased in HO-1 siRNA/FSS-treated NP cells compared to those in control siRNA/FSS-treated cells, which was reversed by CoPP treatment (Figures 3K,L).

### Rapamycin Reverses HO-1 Knockdown-Induced Alterations in Autophagy and ECM Homeostasis in FSS-Treated NP Cells

**FIGURE 4:**
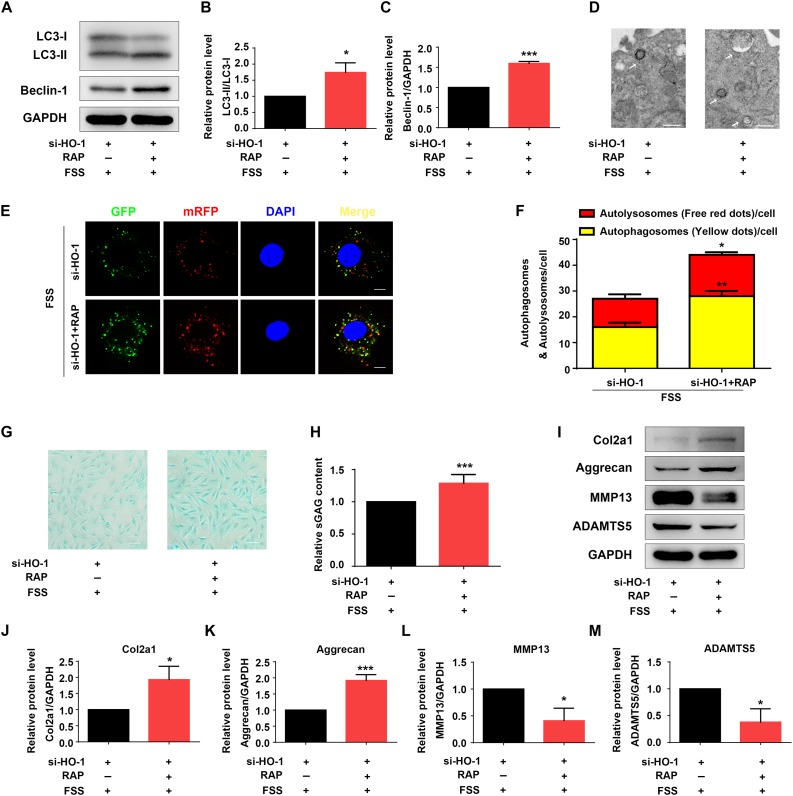
Rapamycin regulates autophagy and ECM in NP cells. **(A–C)** Western blotting. NP cells were first transfected with HO-1 siRNA and treated with autophagy activator rapamycin (RAP, 500 nM) for 12 h. Cells were then treated with 12 dyne/cm^2^ FSS for 2 h, followed by western blotting for expression of the indicated proteins. Quantification of LC3-II/LC3-I ratio and Beclin-1 from three independent experiments **(B,C)**. **(D)** Transmission Electron Microscopy (TEM). NP cells were treated as in panel **(A)**. TEM images are shown. The arrows indicate double-membrane autophagosomes and autolysosomes. Bar, 500 nm. **(E,F)** Measurements of autophagosomes and autolysosomes. NP cells were transduced with lentivirus expressing a tandem mRFP-GFP-LC3 construct and treated as in panel **(A)**. Fluorescence images were obtained. Bar, 5 μm. Quantification of autophagosomes (yellow dots) and autolysosomes (free red dots) from three independent experiments **(F)**. **(G,H)** sGAG content. NP cells were treated as in panel **(A)**, followed by alcian blue staining **(G)** and Blyscan Sulfated Glycosaminoglycan Assay **(H)**. Bar, 100 μm. **(I–M)** Western blotting. NP cells were treated as in panel **(A)**, followed by western blotting for expression of the indicated proteins **(I)**. Quantification of Col2a1, aggrecan, MMP13 and ADAMTS5 from three independent experiments **(J–M)**. Results are expressed as mean ± standard deviation (s.d.). *P < 0.05 and ***P < 0.001 vs. FSS + HO-1-siRNA group.

Rapamycin activates autophagy in multiple cell types (Spang et al., 2014; Lopez de Figueroa et al., 2015). We next determined the effect of rapamycin treatment on autophagy suppression induced by HO-1 knockdown in FSS-treated NP cells. NP cells were first transfected with HO-1 siRNA and then pretreated with or without rapamycin for 12 h and subjected to FSS treatment for 2 h. The results showed that rapamycin treatment of the NP cells significantly increased the LC3-II/LC3-I ratio, expression level of Beclin-1 protein and formation of autophagosome and autolysosome (Figures 4A–F). Furthermore, rapamycin treatment also reversed the decrease of sGAG content and alterations in expression of ECM proteins induced by HO-1 knockdown in the FSS-treated NP cells (Figures 4G–M).

### Primary Cilium Protein IFT88 Is Critical for HO-1 Regulation of Autophagy and ECM Homeostasis in FSS-Treated NP Cells

**FIGURE 5:**
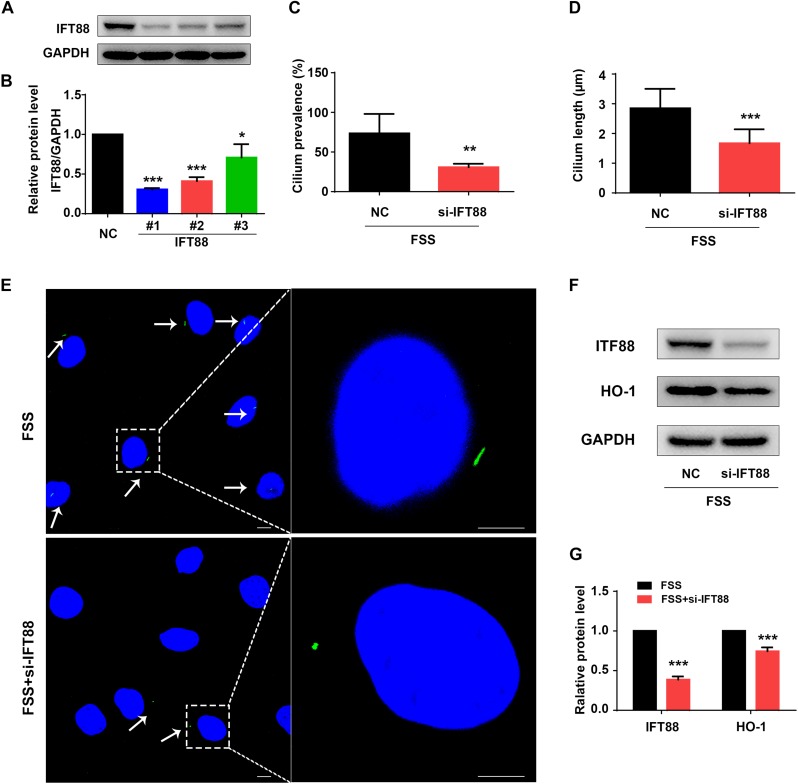
Knockdown of IFT88 impairs cilium function and decreases expression of HO-1 in FSS-treated NP cells. **(A)** IFT88 Knockdown. NP cells were transfected with negative control siRNA (NC-siRNA) and three different IFT88-siRNA (#1, #2, #3). Then, cells were subjected to western blotting for expression of IFT88 **(A)**. Quantitative data from three independent experiments **(B)**. The #1 IFT88-siRNA was used for the following experiments in this study. **(C,D)** Qualification of cilium prevalence **(C)** and cilium length **(D)** in NP cells from three independent experiments. NP cells were transfected with negative control siRNA (NC-siRNA) and IFT88 siRNA. Then, cells were treated with 12 dyne/cm^2^ FSS for 2 h. **(E)** Fluorescence images of primary cilia. Left bar, 10 μm; right bar, 5 μm. White arrows represent primary cilia. **(F,G)** Western blotting. NP cells were transfected with NC-siRNA or IFT88-siRNA (#1) and subjected to 12 dyne/cm^2^ FSS for 2 h, followed by western blotting for expression of IFT88 and HO-1 **(F)**. Quantification of data from three independent experiments **(G)**. Results are expressed as mean ± standard deviation (s.d.). *P < 0.05, **P < 0.01, and ***P < 0.001 vs. NC or FSS + NC-siRNA group).

**FIGURE 6:**
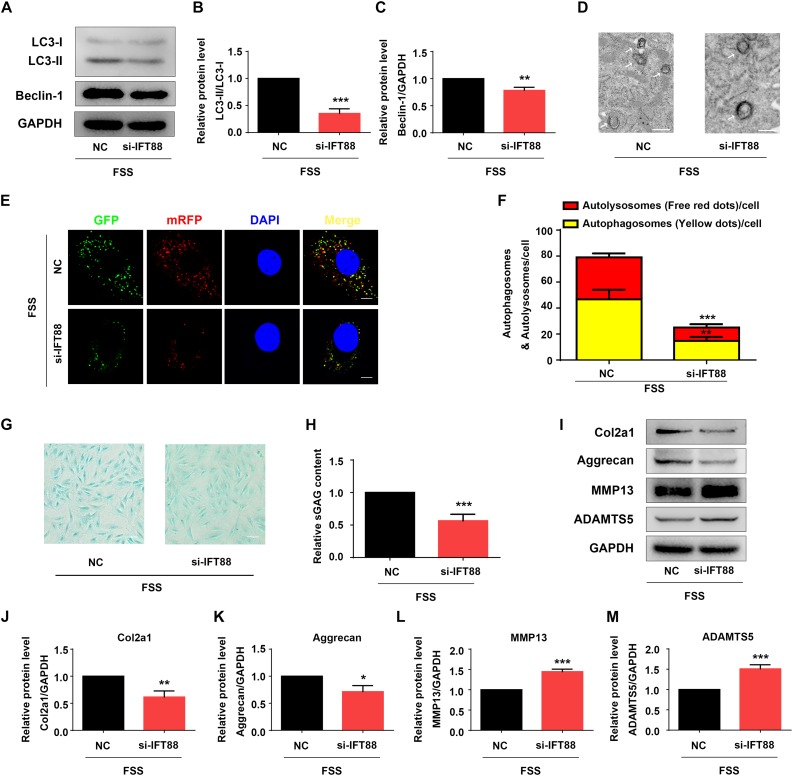
IFT88 loss reduces autophagy and impairs ECM homeostasis in FSS-treated NP cells. **(A–C)** Western blotting. NP cells were transfected with negative control siRNA (NC-siRNA) or IFT88-siRNA (#1). Then, cells were subjected to 12 dyne/cm^2^ FSS for 2 h, followed by western blotting for expression of LC3-I, LC3-II, and Beclin-1 **(A)**. Quantification of LC3-I/LC3-II ratio and Beclin-1 from three independent experiments **(B,C)**. **(D)** Transmission Electron Microscopy (TEM). NP cells were treated as in PANEL **(A)**. TEM images are shown. The arrows indicate double-membrane autophagosomes and autolysosomes. Bar, 500 nm. **(E,F)** Measurements of autophagosomes and autolysosomes. NP cells were transduced with lentivirus expressing a tandem mRFP-GFP-LC3 construct and treated as in panel **(A)**. Fluorescence images were obtained. Bar, 5 μm. Quantification of autophagosomes (yellow dots) and autolysosomes (free red dots) from three independent experiments **(F)**. **(G,H)** sGAG content. NP cells were treated as in panel **(A)**, followed by alcian blue staining **(G)** and Blyscan Sulfated Glycosaminoglycan Assay **(H)**. Bar, 100 μm. **(I–M)** Western blotting. NP cells were treated as in panel **(A)**, followed by western blotting for expression of the indicated proteins **(I)**. Quantification of expression of Col2a1, aggrecan, MMP13 and ADAMTS5 from three independent experiments **(J–M)**. Results are expressed as mean ± standard deviation (s.d.). *P < 0.05, **P < 0.01, and ***P < 0.001 vs. FSS + NC-siRNA group.

Primary cilium was reported to function as a mechano-sensor and plays an important role in mechanotransduction (He et al., 2016). As a core trafficking protein of primary cilium, IFT88 is critical for ciliary assembly and function (Coveney et al., 2018; Fu et al., 2019). We used IFT88-siRNA to knock down its expression in NP cells (Figures 5A,B). The #1 IFT88 siRNA displayed the best knocking down efficiency and was used in the following experiments (Figures 5A,B). Results showed that IFT88 knockdown significantly reduced the cilium prevalence and cilium length in FSS-treated NP cells (Figures 5C–E). Interestingly, IFT88 knockdown decreased the protein level of HO-1 in FSS-treated NP cells (Figures 5F,G). Moreover, IFT88 knockdown inhibited the autophagy activation by decreasing the LC3-II/LC3-I ratio, expression of Beclin-1 protein and formation of autophagosome and autolysosome in FSS-treated NP cells (Figures 6A–F). Furthermore, IFT88 knockdown decreased sGAG content and expression of anabolic proteins (Col2a1 and aggrecan) and increased expression of catabolic proteins (MMP13 and ADAMTS5) (Figures 6G–M).

### CoPP or Rapamycin Largely Reverses IFT88 Loss-Induced Alterations in Autophagy and ECM Homeostasis in FSS-Treated NP Cells

**FIGURE 7:**
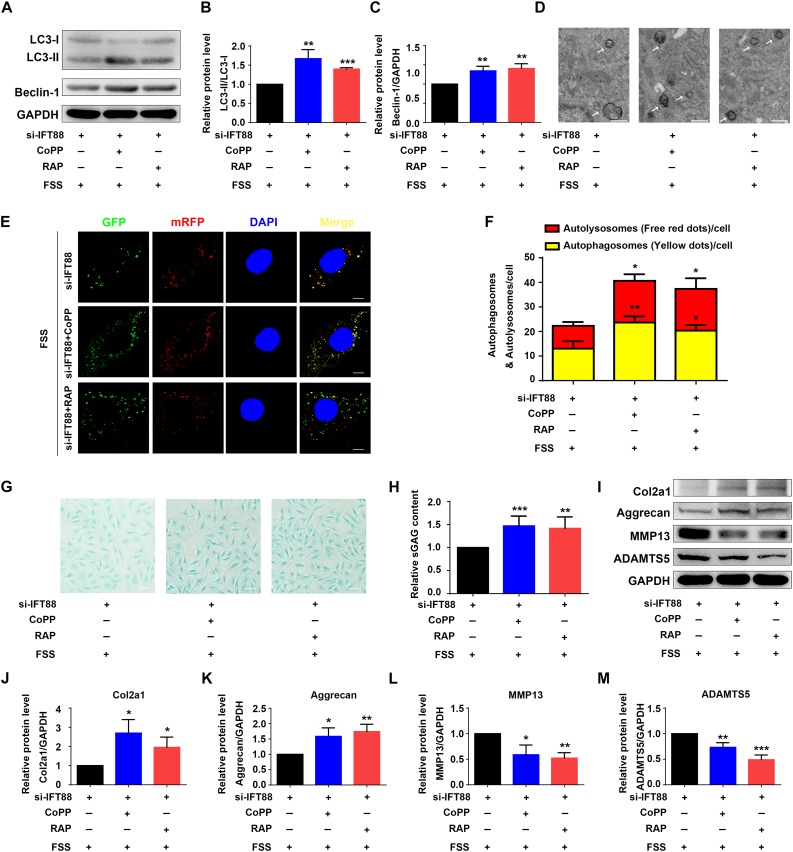
HO-1 upregulation or rapamycin reverses IFT88 loss-induced alterations in autophagy and ECM production in FSS-treated NP cells. **(A–C)** Western blotting. NP cells were transfected with IFT88-siRNA. Then, cells were pretreated with CoPP (10 μM) or rapamycin (RAP, 500 nM) and subjected to 12 dyne/cm^2^ FSS for 2 h, followed by western blotting for expression of LC3-I, LC3-II, and Beclin-1 **(A)**. Quantification of LC3-I/LC3-II ratio and Beclin-1 from three independent experiments **(B,C)**. **(D)** Transmission Electron Microscopy (TEM). NP cells were treated as in panel **(A)**. TEM images are shown. The arrows indicate double-membrane autophagosomes and autolysosomes. Bar, 500 nm. **(E,F)** Measurements of autophagosomes and autolysosomes. NP cells were transduced with lentivirus expressing a tandem mRFP-GFP-LC3 construct and treated as in panel **(A)**. Fluorescence images were obtained. Bar, 5 μm. Quantification of autophagosomes (yellow dots) and autolysosomes (free red dots) from three independent experiments **(F)**. **(G,H)** sGAG content. NP cells were treated as in panel **(A)**, followed by alcian blue staining **(G)** and Blyscan Sulfated Glycosaminoglycan Assay **(H)**. Bar, 100 μm. **(I–M)** Western blotting. NP cells were treated as in panel **(A)**, followed by western blotting for expression of the indicated proteins **(I)**. Quantification of expression of Col2a1, aggrecan, MMP13 and ADAMTS5 from three independent experiments **(J–M)**. Results are expressed as mean ± standard deviation (s.d). *P < 0.05, **P < 0.01, and ***P < 0.001 vs. FSS + IFT88-siRNA group.

Finally, we determined whether rapamycin or upregulation of HO-1 by CoPP can reverse the alterations in autophagy and ECM protein expression induced by IFT88 knockdown in FSS-treated NP cells. NP cells were first transfected with IFT88-siRNA. Then, cells were pretreated with CoPP (10 μM) or rapamycin (500 nM) and then subjected to FSS treatment. The results showed that both CoPP or rapamycin treatment largely reversed the IFT88 knockdown-induced alterations in LC3-II/LC3-I ratio and Beclin-1 protein level (Figures 7A–C), formation of autophagosome and autolysosome (Figures 7D–F), sGAG content (Figures 7G,H) and expression of anabolic and catabolic ECM proteins (Figures 7I–M).

## Discussion

In the present study, we demonstrate the effect of FSS on the regulation of ECM homeostasis in NP cells. We find that FSS regulates ECM metabolism in a loading magnitude- and time-dependent manner. When NP cells are subjected to the 24 dyne/cm^2^ FSS treatment, the balance between ECM anabolism and catabolism is disrupted, as demonstrated by decreases in sGAG content and expression of Col2 and aggrecan and increase in expression of ECM-degrading proteinases MMP13 and ADAMTS5. However, when NP cells are exposed to the relatively moderate FSS (12 dyne/cm^2^ for 1–2 h), FSS maintains the ECM homeostasis by promoting the ECM anabolism and inhibiting the ECM catabolism. The observed pro-anabolism and anti-catabolism effects of moderate FSS are consistent with previous studies in various cell types exposed to different mechanical stimuli. In rat fibrochondrocytes, Deschner et al. (2006) reported that dynamic tensile strain could significantly abrogate IL-1β-induced upregulation of MMPs (MMP3, MMP7, MMP8, MMP9, MMP13, MMP16, MMP17, and MMP19). In human chondrocytes, He et al. (2016) found that moderate cyclic tensile strain could reduce the protein expressions of MMP-1 and MMP-13 and exert an anti-catabolism effect to protect cartilage integrity. Results from Dai et al. (2014) demonstrated that dynamic compression could promote the synthesis of Col2 and aggrecan in adipose-derived stem cells. Our results suggested that NP cells could start an early ECM synthesis process to maintain ECM homeostasis in response to moderate FSS, and a deeper understanding of the specific mechanism by which moderate FSS regulates ECM homeostasis in NP cells might help us to seek for novel and early preventive and therapeutic targets for IVD degeneration.

Increasing evidence have revealed that HO-1 could attenuate inflammation- and oxidative stress-induced imbalance between ECM anabolism and catabolism in NP cells and chondrocytes (Guillen et al., 2008; Hu et al., 2016; An et al., 2018). In this study, we performed RNA sequencing analysis and focused on HO-1, which was significantly upregulated in NP cells exposed to moderate FSS. We found that HO-1 inhibition decreased sGAG content, downregulated ECM anabolism related proteins (Col2a1, aggrecan) and upregulated ECM catabolism related proteins (MMP13, ADAMTS5) in NP cells exposed to moderate FSS, which could be reversed by HO-1 inducer CoPP. These results suggest that HO-1 plays a critical role in the maintenance of ECM homeostasis induced by moderate FSS.

Results from different research groups indicated that FSS could induce protective autophagy in osteocytes, chondrocytes and hepatocellular carcinoma cells (Wang et al., 2018; Zhang et al., 2018; Yang et al., 2019). In line with previous studies, our data demonstrated that moderate FSS activated autophagy in NP cells by increasing the ratio of LC3-II to LC3-I, the protein expression of Beclin-1 and the number of autophagosomes and autolysosomes. HO-1 upregulation as a mean for autophagy induction has been reported in many diseases (Nakamura et al., 2018; Vasconcellos et al., 2018). For example, in a cadmium-induced emphysema mice model, Surolia et al. (2015) found that HO-1 mediated the activation of autophagy and protected against pulmonary endothelial cell apoptosis and development of emphysema. And in a diabetic nephropathy cell model, Dong et al. (2015) reported that HO-1 could enhance autophagy and inhibited high glucose-induced podocytes apoptosis. In this study, we demonstrated that HO-1 inhibition suppressed the FSS-induced autophagy activation, while HO-1 inducer CoPP reversed this process. Furthermore, autophagy activator rapamycin attenuated HO-1 inhibition induced disruption of FSS-induced autophagy activation and ECM homeostasis in NP cells. Collectively, these results suggest that moderate FSS maintains ECM homeostasis in NP cells, at least in part, through HO-1-mediated autophagy.

**FIGURE 8:**
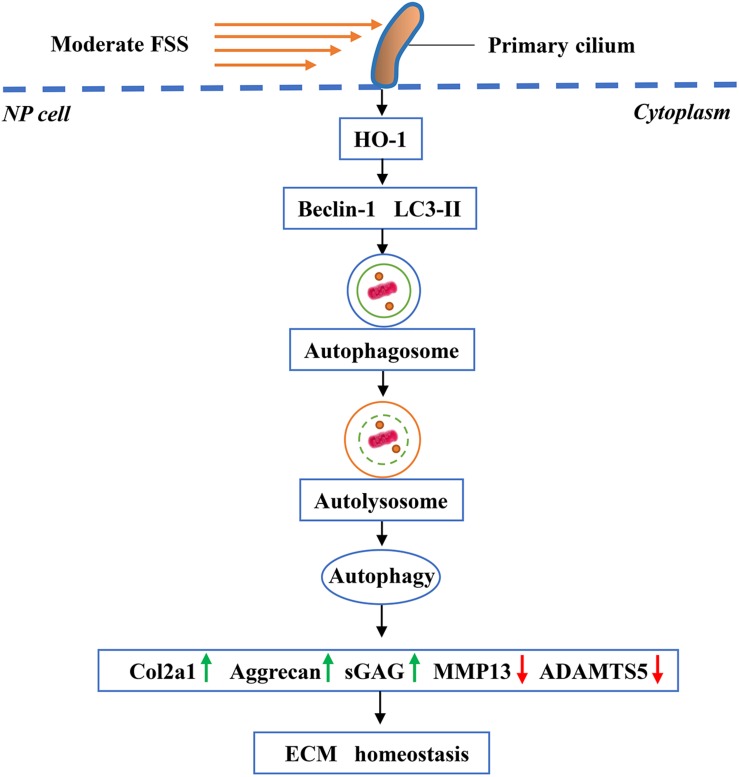
Working model. Primary cilia sense and transmit extracellular FSS signal to NP cells, which activates HO-1 and thereby autophagy. Increased autophagy plays an important role in maintaining ECM homeostasis in NP cells.

Although our results above revealed that FSS could regulate ECM homeostasis through HO-1-mediated autophagy in NP cells, how the cells sense FSS and convert it into downstream molecular signals remained unclear. Primary cilium is a microtubule-based organelle, which extends as a solitary protrusion from the surface of most mammalian cell types, including NP cells (Zheng et al., 2018; Anvarian et al., 2019). It can sense and transmit extracellular mechanical and chemical signals, to regulate various cellular processes and maintain tissue homeostasis (Hua and Ferland, 2018; Kim and Kim, 2019). Because primary cilium cannot synthesize proteins, cilium-associated trafficking proteins, such as IFT88, are therefore critical for cilium biogenesis, maintenance and function (Wang et al., 2016). Recent studies have demonstrated that hypomorphic mutation of IFT88 can result in severely stunted primary cilia and abolish the suppression effects of cyclic tensile strain on the inflammatory response to IL-1β in chondrocytes (Fu et al., 2019). Results from He et al. (2016) showed that disruption of the primary cilia in chondrocytes by IFT88-siRNA attenuated the anti-catabolism effects of moderate cyclic tensile strain. Moreover, Orhon et al. (2016) reported that hypomorphic deletion of the IFT88 impaired ciliary assembly and inhibited the FSS-induced autophagy activation in epithelial cells. Hence, we speculated that primary cilia associated with IFT88 might play a pivotal role in the mechanotransduction when NP cells were exposed to FSS. As expected, NP cells transfected with IFT88-siRNA exhibited defective primary cilia, and the disruption of primary cilia downregulated the protein expression of HO-1, inhibited the autophagy activation and destroyed the ECM homeostasis in NP cells exposed to moderate FSS. Meanwhile, we found that both CoPP and RAP partially reversed the changes. Therefore, our data suggest that primary cilium could sense and mediate moderate FSS signal to regulate HO-1 mediated autophagy activation and then maintain ECM homeostasis in NP cells (Figure 8).

## Conclusion

Findings from our study indicate that moderate FSS could maintain ECM homeostasis in NP cells which requires HO-1-mediated autophagy activation in the presence of primary cilium. This study could lead us to a better understanding of moderate FSS-mediated maintenance of ECM homeostasis in NP cells, and may shed lights into developing novel strategies for early prevention and treatment of IVD degeneration.

## Data Availability Statement

The RNASeq data have been deposited in Sequence Read Archive (SRA, PRJNA587407).

## Author Contributions

GX, ZS, SC, and HC designed the study. SC, HC, LQ, XW, XF, and SL conducted the study and collected the data. SC and GX analyzed the data. GX, ZS, SC, DC, and HC interpreted the data. SC, HC, and GX drafted the manuscript. SC, GX, LQ, XW, XF, SL, ZS, and HC took the responsibility for the integrity of the data analysis.

## Conflict of Interest

The authors declare that the research was conducted in the absence of any commercial or financial relationships that could be construed as a potential conflict of interest.

## Abbreviations

ADAMTS5: A disintegrin and metalloproteinase with thrombospondin motif 5
AF: annulus fibrosus
CEP: cartilage endplate
Col2: collagen type II
CoPP: cobalt protoporphyrin IX
CS: chondroitin sulfate
ECM: extracellular matrix
FSS: fluid shear stress
HA: hyaluronic acid
HO-1: heme oxygenase-1
IFT: intraflagellar transport
IVD: intervertebral disk
KS: keratin sulfate
LBP: low back pain
MMP13: matrix metalloproteinase 13
NP: nucleus pulposus
PGs: proteoglycans
RAP: rapamycin
sGAG: sulfated glycosaminoglycan.
