# Supplementary material for: Moderate Fluid Shear Stress Regulates Heme Oxygenase-1 Expression to Promote Autophagy and ECM Homeostasis in the Nucleus Pulposus Cells
Source: Front Cell Dev Biol. 2020 Mar 3;8:127. doi: 10.3389/fcell.2020.00127 (PMC7064043; doi:10.3389/fcell.2020.00127)
Supplement: Supplementary file 1 [file Table_1.DOCX]

**Supplementary Table 1** Primer sequences used in qRT-PCR and siRNA sequences used in siRNA transfection

| **Gene** | **Forward (5’-3’)** | **Reverse (5’-3’)** |
| --- | --- | --- |
| HO-1 | AAGCCGAGAATGCTGAGTTCA | GCCGTGTAGATATGGTACAAGGA |
| GAPDH | AGGTCGGTGTGAACGGATTTG | TGTAGACCATGTAGTTGAGGTCA |
|  | | |
| **siRNA** | **Sense (5’-3’)** | **Antisense (5’-3’)** |
| NC | UUCUCCGAACGUGUCACGUTT | ACGUGACACGUUCGGAGAATT |
| HO-1 #1 | CAGGAAAUCAUCCCUUGCATT | UGCAAGGGAUGAUUUCCUGTT |
| HO-1 #2 | CAGAUCAGCACUAGCUCAUTT | AUGAGCUAGUGCUGAUCUGTT |
| HO-1 #3 | GUAAGGGAGAAUCUUGCCUTT | AGGCAAGAUUCUCCCUUACTT |
| IFT88 #1 | GGACUUAACCUACUCCGUUTT | AACGGAGUAGGUUAAGUCCTT |
| IFT88 #2 | CCAACGACCUGGAGAUUAATT | UUAAUCUCCAGGUCGUUGGTT |
| IFT88 #3 | GCUUGGAGCUUAUUACAUUTT | AAUGUAAUAAGCUCCAAGCTT |
