# Supplementary material for: Moderate Fluid Shear Stress Regulates Heme Oxygenase-1 Expression to Promote Autophagy and ECM Homeostasis in the Nucleus Pulposus Cells
Source: Front Cell Dev Biol. 2020 Mar 3;8:127. doi: 10.3389/fcell.2020.00127 (PMC7064043; doi:10.3389/fcell.2020.00127)
Supplement: Supplementary file 3 [file Image_1.pdf]

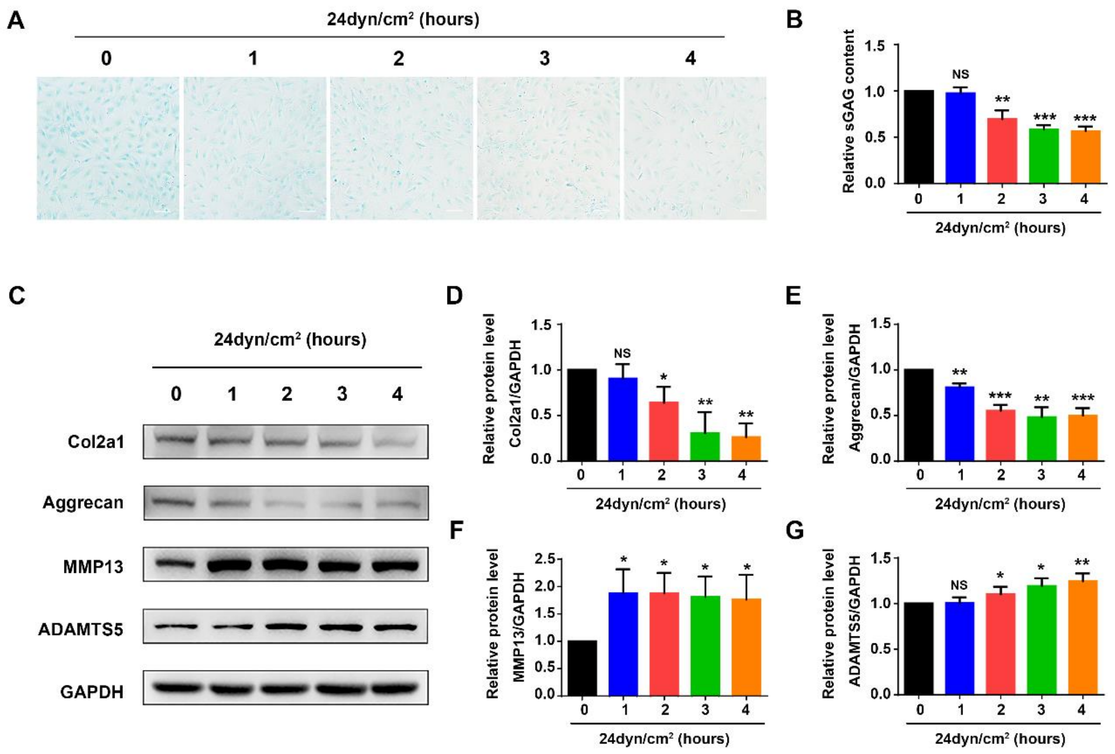

Supplementary Figure 1 FSS of 24 dyne/cm^2 regulated ECM metabolism. **a** Alcian blue staining for NP cells stimulated with FSS for indicated times (0, 1, 2, 3 and 4 hours). **b** Relative sGAG content of NP cells detected by Blyscan Sulfated Glycosaminoglycan Assay. **c** The typical western blot bands of Col2a1, aggrecan, MMP13 and ADAMTS5. **d-g** Summary data showing protein levels of Col2a1, aggrecan, MMP13 and ADAMTS5. NS means no statistical significant difference. The data are expressed as mean ± SD from three independent experiments. (*P < 0.05, **P < 0.01 and ***P < 0.001 vs. the group of 0 hour group)
